# Breakfast Consumption Habit and Its Nutritional Contribution in Latin America: Results from the ELANS Study

**DOI:** 10.3390/nu12082397

**Published:** 2020-08-10

**Authors:** Mauro Fisberg, Irina Kovalskys, Agatha Nogueira Previdelli, Jaqueline Lopes Pereira, Ioná Zalcman Zimberg, Regina Fisberg, Gerson Ferrari, Viviana Guajardo, the ELANS Study Group

**Affiliations:** 1 Instituto Pensi, Fundação Jose Luiz Egydio Setubal, Hospital Infantil Sabara, Av. Angelica 1968, conjs 71 a 74, São Paulo 01239-040, Brazil; 2 Departamento de Pediatria, Universidade Federal de São Paulo, Rua Botucatu, 598, Vila Clementino, São Paulo 04023-062, Brazil; 3 Facultad de Ciencias Médicas, Pontifica Universidad Católica (UCA), Av. Alicia Moreau de Justo 1300, Buenos Aires C1107AAZ, Argentina; ikovalskys@gmail.com (I.K.); vbguajardo@gmail.com (V.G.); 4 Faculdade de Ciências Biológicas e da Saúde, Universidade São Judas Tadeu, Rua Taquari, 546, Mooca, São Paulo 03166-000, Brazil; agatha.usp@gmail.com; 5 Departmento de Nutrição, Faculdade de Saúde Pública, Universidade de São Paulo (USP), São Paulo 01246-904, Brazil; jaque.lps@gmail.com (J.L.P.); regina.fisberg@gmail.com (R.F.); 6 Departamento de Psicobiologia, Universidade Federal de São Paulo, Rua Botucatu, 862, Vila Clementino, São Paulo 04023062, Brazil; iona.zimberg@gmail.com; 7 Laboratorio de Ciencias de la Actividad Física, el Deporte y la Salud, Facultad de Ciencias Medicas, Universidad de Santiago de Chile, USACH, Santiago 7500618, Chile; gersonferrari08@yahoo.com.br

**Keywords:** breakfast, nutritional intake, cross-sectional study, Latin America

## Abstract

The aim of this study was to provide updated data on breakfast consumption, associated factors and its contribution to daily intakes among Latin American populations. A total of 9218 subjects, 15 to 65 years old, were evaluated in the ELANS study, a multicenter cross-sectional study conducted in eight Latin American countries (Argentina, Brazil, Chile, Colombia, Costa Rica, Ecuador, Peru and Venezuela). Dietary data were obtained by two 24 h dietary recalls. Overall, 78.6% of the population were regular breakfast consumer, 15.9% occasional and 5.5% skippers. Adolescents were found to be the most frequent occasional consumers (19.2%) and skippers (6.8%). Among breakfast consumers (*n* = 8714), breakfast contributed to 444 ± 257 kcal, i.e., 23% of the total daily EI (16–27%). Breakfast consumers were more likely to be older adults than adolescents (OR = 1.49, 95% CI:1.06–2.10) and physically active than insufficiently active (OR = 1.29, 95% CI:1.07–1.55), and were less likely to be underweight than normal weight (OR = 0.63, 95% CI:0.41–0.98). In most countries, breakfast was rich in carbohydrates, added sugars, saturated fat and calcium relative to the entire day, and the energy contribution of protein and fats was lower at breakfast than for the entire day. These findings will contribute to the development of data-driven nutrient recommendations for breakfast in Latin America.

## 1. Introduction

Over the centuries breakfast has been recognized across the world as an essential meal. A considerable body of scientific evidence from both randomized controlled trials and observational studies indicates the relevancy of breakfast consumption and its associated contributions to health [1,2] and nutrient-related outcomes [3,4,5].

Despite its importance, the dietary habit of skipping breakfast is quite frequent among countries, with reports showing 4–24% of adults [6,7,8,9,10,11] and 4–26% of adolescents [6,7,8,9,10,11] frequently skipping the morning meal. Of considerable importance is the association between breakfast skipping, unhealthy eating behavior and health outcomes [12]. Many studies have demonstrated positive associations between omitting breakfast and increased bodyweight [13,14], cardiovascular disease [15,16,17], dyslipidemia [18] and type 2 diabetes mellitus [19].

Regular breakfast consumption has been associated with higher intake of healthy foods such as milk, fruits, vegetables and grains, rather than foods high in calories, fats and sugar [20], higher daily intake of micronutrients [4,21,22] and higher tendency to meet nutritional recommendations [5].

Several studies have consistently reported a wide variation in the contribution of breakfast to nutrient intake and diet quality in various parts of the world [6,7,8,9,10,11]. The International Breakfast Research Initiative (IBRI) was launched in 2016 as the first transatlantic international collaborative study to assess the breakfast consumption of representative dietary surveys of six different countries (Canada, Denmark, France, Spain, United Kingdom, United States of America) using a standard breakfast definition [23]. Within this context and considering the lack of studies that have provided detailed data on the Latin American (LA) dietary composition of breakfast or that have examined the differences across countries and among regions, the recent Latin American Study of Nutrition and Health/Estudio Latinoamericano de Nutrición y Salud (ELANS) study has joined the IBRI consortium.

The purpose of the current study is to provide updated data on the breakfast consumption habit and to explore the factors potentially associated with breakfast consumption, its nutritional composition and contribution to daily intake among the LA population.

## 2. Material and Methods

### 2.1. Data Source and Study Sample

Data from this study were drawn from the ELANS study, a multicenter cross-sectional study carried out in eight Latin American countries (Argentina, Brazil, Chile, Colombia, Costa Rica, Ecuador, Peru and Venezuela). Dietary intake and physical activity status of household-based individuals were evaluated simultaneously over a period of one year (September 2014 to August 2015) and only representative samples from urban populations, where 80–90% of the population of these countries is living, were randomly recruited.

The final sample was comprised of 9218 subjects from 15 to 65 years old. It was a random complex multistage sample, designed to be representative in terms of geographical region, sex, age and socioeconomic level (SEL), with a random selection of Primary Sampling Units (PSU) and Secondary Sampling Units (SSU). Within each SSU, households were selected through systematic randomization and selection of respondent within a household using the birthday method (50% of the sample chosen using the next birthday and 50% using the last birthday), with quotas controlled for sex, age and SEL. The sampling size was calculated with a confidence level of 95% and a maximum error of 3.49%. Based on guidance from the U.S. National Center for Health Statistics [24], a study design effect of 1.75 was estimated and minimum sample sizes required per strata (i.e., SEL, age and sex) were calculated for each country. Sample weighting was computed for each individual country level considering key variables of interest (the geographical region, sex, age and SEL). Sample weighting was not applied on the unified (eight countries) database given the lack of an official publication of the LA urban population distribution. All analyses presented in the current paper were performed using this unified database.

The overarching ELANS protocol was approved by the Western Institutional Review Board (#20140605) and has been registered at Clinical Trials (#NCT02226627). Ethical review boards of each participating institutions have also approved the protocol. All of the study sites performed simultaneously a common study protocol for training of interviewers, implementation of fieldwork, data collection and management and quality control procedures. Informed consent/assent was given by all participants before participation in the survey. Detailed information on this study design, protocol and methodology has been previously published [25].

### 2.2. Dietary Assessment

Dietary data were obtained using two non-consecutive 24 h dietary recalls (24-HR) according to the Multiple Pass Method [26], which provided detailed information on all food and beverages, including water and alcoholic beverages, preparations/recipes and supplements consumed over the 24 h previous to the interview. In order to capture the day-to-day variation intake, both weekdays and weekend days, with a proportional distribution of days among the sample, were included in the 24-HR. Household measures and a photographic album containing the most common household utensils and portion sizes adapted to each country were used to quantify reported intakes. Each food/recipe was transformed into grams and milliliters by trained nutritionists and then analyzed as energy, macronutrients and micronutrients using the Nutrition Data System for Research software, versions 2013 (for Argentina, Chile, Costa Rica, Ecuador and Venezuela) and 2014 (for Brazil, Colombia, and Peru) (NDS-R, Minnesota University, MN, USA). A nutritional equivalency of local and traditional food items to foods available in the USDA composition table of the NDS-R software database was performed through a comprehensive process in each country. Professional nutritionists of each country conducted a food matching standardized procedure during the data entry in order to minimize errors and verify quantities of key nutrients. The complete procedure for standardization of the food composition database has been described in detail elsewhere [27].

Estimates of usual intake for each nutrient were determined with the National Cancer Institute (NCI) method [28], which accounts for within- and between-individual variance components and corrects for the high intraindividual variation intrinsic to 24-HR. Due to differences in eating habits among the Latin American populations, the estimation of usual intake was conducted separately for each country.

### 2.3. Definition of Breakfast

Breakfast was self-reported by the respondent and included consumption of any food or beverage of at least 50 kcal at a meal occasion named as breakfast. A total of 18,436 24-HR (i.e., 9218 individuals x two 24-HR = 18,436) were assessed. The selection of breakfast eaters occurred in four stages: (1) Self-report of breakfast. As the 24HR assessment followed the Multiple Pass Method [26] the individuals had to report the meal occasion name during the assessment [25,26]. In this stage, of 18,436 recalls collected, 16,699 self-reported breakfast. (2) A consistency analysis to determine plausible breakfast energy intake (EI) of at least 50 kcal [29]. In this stage, 731 recalls were excluded. (3) Breakfasts with EI derived exclusively from alcoholic beverages were not included in the final sample. In this stage, 6 recalls were excluded. (4) A consistency analysis of EI contribution of breakfast in the 24-HR to identify potential typing errors. In this stage 15 recalls were reviewed, 4 were adjusted and 1 excluded (Supplementary Materials Figure S1).

Regularity of breakfast consumption was defined as follows: regular breakfast consumers, who consumed breakfast on both dietary assessment days; occasional breakfast consumers, who consumed breakfast only on one day; and breakfast skippers, who did not consume breakfast on both days. The final sample comprised 504 breakfast skippers, 1467 occasional breakfast consumers and 7247 regular breakfast consumers.

### 2.4. Anthropometric, Sociodemographic and Physical Activity Data

All participating countries performed anthropometric measurements of body weight, height and waist, hip and neck circumferences according to standardized procedures [25]. Body mass index (BMI) in adolescents (15 to 19 years old) was categorized according to the BMI-for-age and sex cut-off points from WHO [30] for underweight (BMI for age < −2 SD), normal weight (−2 SD ≥ BMI for age ≤ 1 SD), overweight (1 SD ≥ BMI for age ≤ 2 SD) and obesity (BMI for age > 2 SD) categories. In adults and older adults (older than 19 years old) BMI was categorized as underweight (<18.5 kg/m^2^), normal weight (18.5–24.9 kg/m^2^), overweight (25–29.9 kg/m^2^) and obesity (≥30.0 kg/m^2^) [31].

Age groups were defined as: adolescents (15 to 19 years old), younger adults (30 to 34 years old), adults (35 to 49 years old) and older adults (50 to 65 years old).

Socioeconomic level (SEL) was evaluated in each country by a country-dependent questionnaire based on the legislative requirements or established local standard layouts. SEL was categorized into three groups (low, medium and high) according to the national indexes used in each country [25].

Physical activity (PA) was assessed using the self-administered version of the Spanish language long-form for the last seven days of the International Physical Activity Questionnaire (IPAQ) [32]. The IPAQ, which has been extensively used in Latin America [32,33,34], contains questions about the amount of walking, moderate and vigorous PA occurring as part of active transport and in leisure time. Only the sections about transport, sitting time and leisure-time PA were included in the ELANS [35]. Data analysis was performed in accordance to the IPAQ scoring protocol (www.ipaq.ki.se) and PA (i.e., leisure-time and transport; min/week) was estimated and used as analysis variables. The proportion of active (i.e., >60 min/day for adolescents and >150 min/week of moderate-to-vigorous PA for adults) or insufficiently active respondents were reported according to the World Health Organization PA guidelines (leisure: walking + moderate + vigorous; transport: walking + bicycle) [35].

### 2.5. Statistical Analysis

Descriptive analyses of mean, percentage and 95% confidence intervals (95% CI) was performed using Stata^®^ software (StataCorp., 2011, Stata Statistical Software: version 12, College Station, TX, USA). These included the prevalence of breakfast consumption by sex, age group, SEL, body weight status, PA and country. Comparison of percentages of the categorized variables was made using the Pearson Chi-square test. A multiple logistic regression model was used to determine the factors associated with breakfast consumption (skipper vs regular/occasional). The analyses considered the following variables: sex, age group, body weight status, SEL and PA among the Latin American population. A *p*-value <0.05 was considered as statistically significant.

Statistical Analysis Software (SAS Institute Inc., Cary, NC, USA, version 9.3) was used to perform MIXTRAN and INDIVINT macros, version 2.1, to predict individual daily nutrient usual intake from two 24-HR [36].

The total intake and proportion of daily energy and nutrients from breakfast were calculated. The contribution of breakfast to daily EI was compared to the recommended value of 20% as proposed by other studies [37,38].

## 3. Results

Overall, most of the participants (78.6%) were regular breakfast consumers while the remainder were either occasional consumers (15.9%) or skippers (5.5%) (Table 1). No significant difference in the proportion of regular breakfast consumers was observed according to sex, SEL or body weight status. According to the age group, older adults (50–65 years) consumed breakfast more frequently (83.2%), followed by adults (35–49 years; 79.9%), young adults (20–34 years; 76.8%) and adolescents (15–19 years; 74.0%; *p* < 0.001). The highest prevalence of occasional consumers and breakfast skippers were found in adolescents (19.2% and 6.8%, respectively), representing one out of four consumers. A higher proportion of breakfast skippers were insufficiently active (6.1%) when compared to active (4.8%; *p* = 0.02).

When we analyzed the factors associated with breakfast intake using a multiple regression model that included sex and SEL as adjustment covariates, it was observed that breakfast consumers were 49% more likely to be older adults than adolescents, 37% less likely to be underweight than normal weight and 29% more likely to be physically active than insufficiently active.

**Figure 1:**
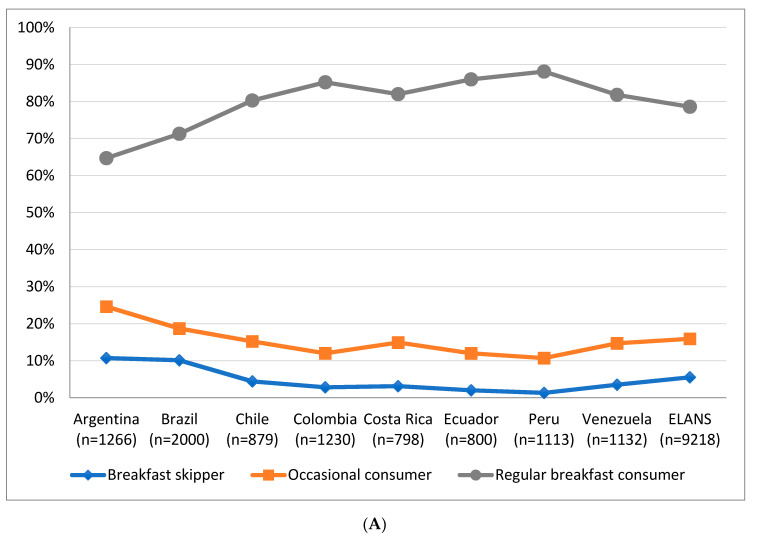

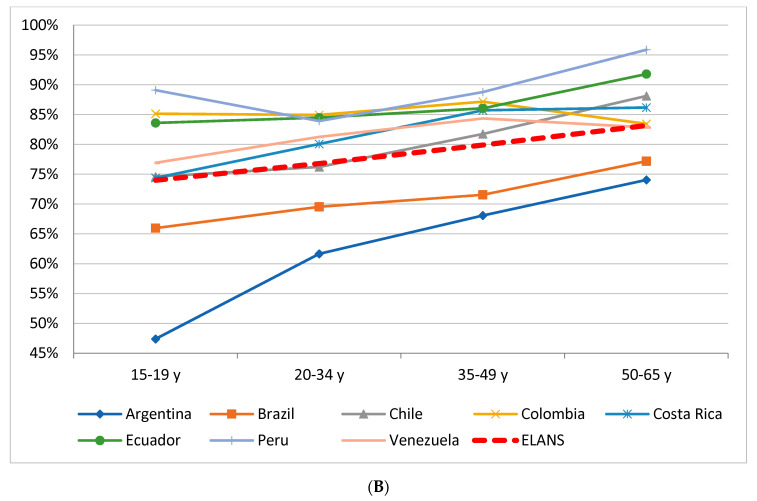
Proportion of regular breakfast consumers (*n* = 7247), occasional consumers (*n* = 1467) and breakfast skippers (*n* = 504) by country (**A**) and proportion of regular breakfast consumers by country and age group (**B**).

Figure 1 shows the large variation in regular breakfast consumers among countries and age groups. Argentina and Brazil were the countries with the highest proportion of breakfast skippers (10.7% and 10.1%, respectively), whereas Peru was the country with the lowest proportion (1.3%) (*p* < 0.001). In Argentina, Brazil, Chile, Costa Rica, Ecuador and Venezuela, a lower proportion of regular consumers among adolescents with an increased proportion throughout the adult age groups was observed. However, the same trend was not observed in Peru, where older adults were followed by adolescents for higher proportion of regular intake, and Colombia, where a lower proportion of regular intake was observed in the older adults group.

The mean intake and percent contribution of breakfast to total daily energy and nutrient intakes among occasional and regular breakfast consumers is shown in Table 2. On average, the ELANS sample consumed about 444 kcal at breakfast, which accounted for 23% of the total daily EI (ranging between 16% and 27% across countries). Argentina’s breakfast contribution to daily EI was the lowest among ELANS countries (15.7%) and therefore the breakfast in this country provided a low percentage of the daily intake of most nutrients. Venezuela’s breakfast contribution to daily EI was the highest (27.3%) among the ELANS countries and therefore it was an important contributor to the daily intake of nutrients in this country.

Overall, breakfast was responsible for ¼ of the daily intake of carbohydrates and fats and 1/5 of the daily intake of proteins. A large variability in the contribution of fats and proteins from breakfast to the daily intake was observed between countries (fat: 11% in Argentina to 32% in Venezuela; protein: 12% in Argentina to 24% in Colombia), while much less variability was observed for carbohydrates (22% in Argentina to 27% in Venezuela).

The contribution of breakfast to mean daily intakes ranged from >30% of total added sugar, riboflavin and calcium; 20–30% of total saturated fat, fiber, sodium, vitamins A, D, thiamin, niacin, vitamin B12, iron, potassium, magnesium and zinc; and <20% of total vitamins B6 and C.

**Figure 2:**
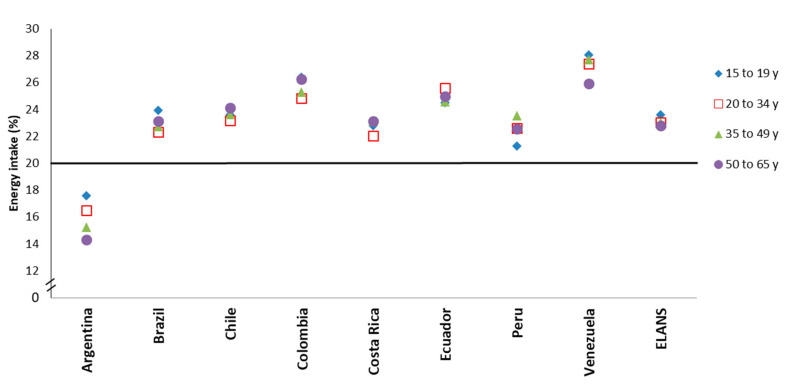
Contribution of breakfast to daily EI (%) among occasional and regular consumers by country. EI: energy intake. The 20% cutoff is indicated by a solid black line.

When examined by age groups (Figure 2), a visual trend on the contribution of breakfast to daily EI across age groups was not observed, except for Argentina, where adolescents had higher %EI and older adults lower.

**Figure 3:**
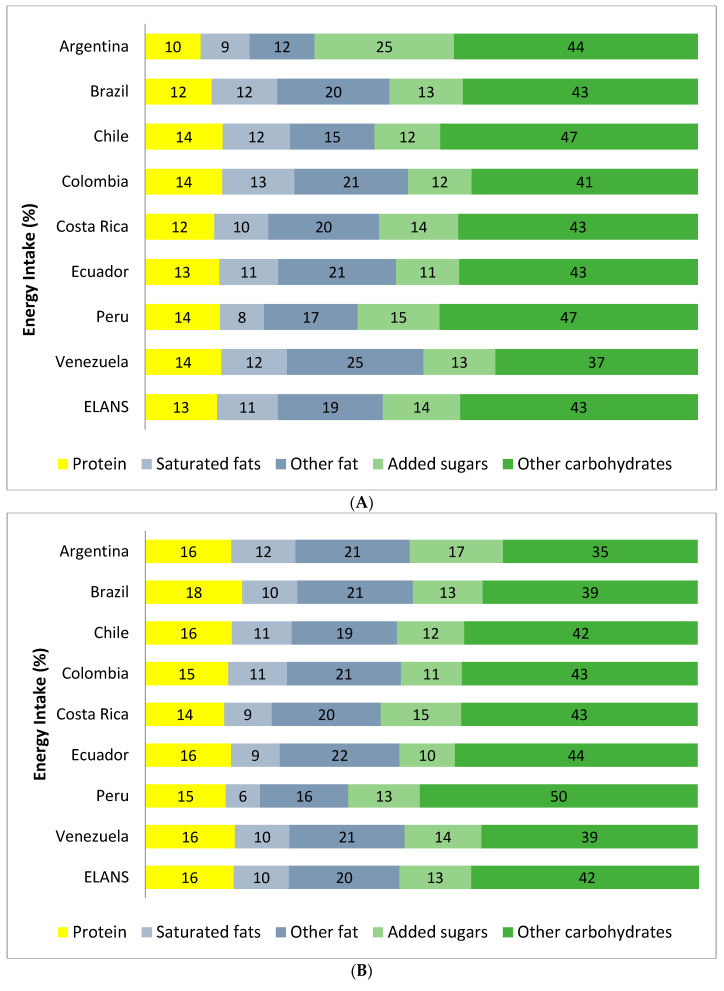

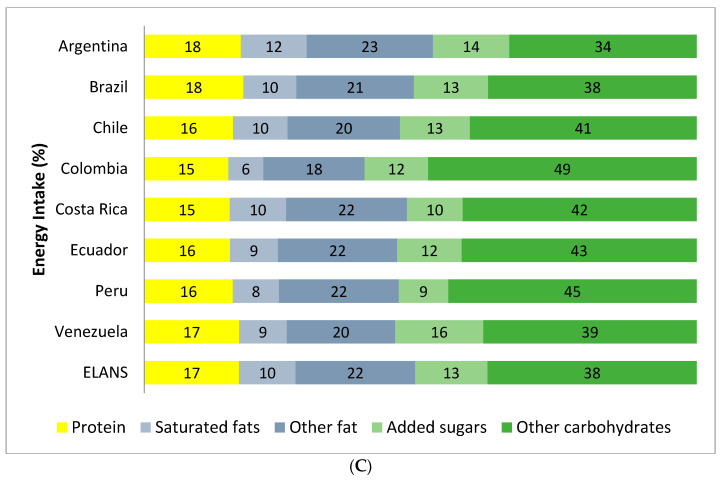
Macronutrient contribution (% energy intake) of breakfast (**A**) relative to the daily intake among occasional and regular breakfast consumers (**B**) and skippers (**C**) by country.

The mean percentage contribution of macronutrients to breakfast and daily EI of breakfast consumers and skippers is shown in Figure 3. For the breakfast consumers sample, the mean contribution of macronutrients to breakfast EI was 57% carbohydrate (total + added sugar), 13% protein, 30% fat (total + saturated), 11% saturated fats and 14% added sugar. As for the total daily EI, this was 55% carbohydrate (total + added sugar), 16% protein, 30% fat (total + saturated), 10% saturated fats and 13% added sugar. As a result, in most countries breakfast was an eating occasion rich in carbohydrates, added sugars and saturated fats relative to the entire day, and the energy contribution of protein and fats was lower at breakfast than for the entire day. Comparing the daily macronutrient intake among breakfast consumers (Figure 3, Panel B) and skippers (Figure 3, Panel C), breakfast skippers had similar mean percentage contribution of macronutrients to daily EI, with a slightly higher intake of other carbohydrates (not added sugar) among breakfast consumers and a slightly higher intake of other fats (not saturated fat) among breakfast skippers.

Argentina and Peru were the countries with the highest percentage of energy derived from carbohydrates (69% and 63%, respectively) when analyzing only the breakfast intake, whereas Peru had the highest percentage when analyzing the total day EI (63%). Venezuela had the lowest percentage of energy from carbohydrates when analyzing only the breakfast intake (50%) and no country had a markedly lower percentage of carbohydrates when analyzing the daily intake. All countries had similar percentage of energy derived from protein (14–16% of breakfast; 10–14% of daily), fat (19–22% breakfast and daily) and saturated fat (9–12% breakfast and daily), except for Peru and Argentina in regards to fats. Argentina had the highest percentage from added sugar both in breakfast and daily EI (25% and 17% respectively).

## 4. Discussion

This first multicentric study with a representative sample of eight Latin American countries provided insights into the breakfast consumption habits, associated factors and the nutritional contribution among Latin Americans living in urban areas. Overall, the majority of the sample reported consuming breakfast (95%; 79% on a regular basis and 16% on an occasional basis), and important differences were observed between countries, age, BMI status and PA level groups.

Breakfast consumption was associated with an older age whereas breakfast skipping (5.5% of sample) was found to be highest among adolescents. This finding is consistent with a body of evidence from several countries [6,7,8,10,11,39] showing that regularity of breakfast intake is commonly lower in adolescents compared to adults of the same populations. Although some studies have hypothesized that this age difference could be a transient phenomenon from a lower frequency of breakfast in adolescents to a regular consumption in adulthood [6], this does not seem to be the case for part of the LA countries assessed in ELANS. In fact, it was observed that Peru had the highest proportion of adolescents and older adults classified as breakfast consumers when compared to the other countries and this explains why Peru was the country with the lowest proportion of breakfast skippers.

Breakfast consumption was also associated with being physically active, which is consistent with many cross-sectional studies [40,41,42]; however, not all studies found such an association [43]. Besides having methodological differences, the majority of these studies have been conducted with children and adolescents, which prevents direct comparisons to the present findings. One possible explanation for the relationship between being physically active and eating breakfast regularly may be that both of these factors are associated with a health-promoting lifestyle [1,2,41,43,44].

In the current study no significant differences were observed among breakfast consumers/skippers and overweight or obesity, and breakfast consumers were less likely to be underweight. This result concurs with previous studies in Brazil, Canada and France that have not observed an association between regular breakfast consumption and prevalence of overweight or obesity [6,9,45]. In two meta-analyses, skipping breakfast was associated with overweight/obesity and increased risk of overweight/obesity [14,46]. Therefore, current evidence linking breakfast consumption and reduced risk of overweight in LA populations remains inconclusive, and reinforces the need for additional research on this topic in the LA region.

A key finding of the current study is that among regular and occasional breakfast consumers, breakfast provided about 23% of daily EI, which is consistent with the range of 20–25% of daily energy that has been suggested as an appropriate intake at breakfast [47]. Breakfast seems to be an important contributor to the daily EI in Venezuela (27.3%), the highest among the ELANS countries, and is markedly low in Argentina (16% of EI). These differences could potentially be related to cultural habits of each country. For example, breakfast in Argentina typically consists of a beverage, such as milk, coffee or tea, combined with solid food (bread, biscuits, pastries, or cereals) [48], whereas in Venezuela breakfast typically consists of a larger variety of foods such as baked corn flour bread called arepas as a side or filled with different proteins and vegetables, corn pancakes filled with cheese, sandwiches or other typical dishes accompanied by juices and/or coffee [49]. These observations highlight the importance of understanding the main foods consumed at breakfast in LA populations in future studies.

At breakfast many nutrients were provided in amounts exceeding 20% of daily intakes, including protein, carbohydrates, fat, saturated fat, total added sugar, fiber, vitamin A, B vitamins (except for B6), vitamin D, calcium, sodium, iron, potassium, magnesium and zinc. In most countries, breakfast was rich in carbohydrates, added sugar, saturated fat and calcium relative to the entire day, and the energy contribution of protein and fats was lower at breakfast than for the entire day. Argentina was the only country which has exceeded the recommendations for total carbohydrates in this meal [50] and this has been explained by the intake of added sugar 2.5 times higher than recommended [51]. Of note, all countries presented an added sugar intake above the recommendation of 10% [51], which corroborates with previous findings from other IBRI studies [6,7,8,11,39] and from ELANS [52,53].

Despite the considerable contribution to the daily intake of micronutrients, overall the LA breakfast was shown to contribute to low intake of vitamin B6 (0.27 mg) and C (18.0 mg) in relation to the current recommended intakes (vitamin B6: 1.1–1.4 mg/d and vitamin C: 60–75 mg/d). Nutrient density was different across the countries, reflecting that food choices may have an impact on the nutrients reported in the breakfast of each country and deserves future investigation.

The relative contribution of breakfast to total nutrient intakes should be approached cautiously as in some cases nutrients with low daily intake presented high relative contribution to breakfast. This occurred for calcium, for example, in which breakfast contributed to about 33% of the total daily intake, and mean daily intake was only 201.7 mg/day, much below the reference of 800 mg/day [54]. In these cases of low total daily intake, breakfast intake should be higher, despite the relatively high contribution, in order to increase daily intakes.

Despite the strengths of the present study, including the use of a standardized methodology with simultaneous application of two individual non-consecutive 24-HR in this large multicentric nationally representative sample of eight LA countries, some limitations should be acknowledged. The findings from the current study should be interpreted with caution because the cross-sectional design cannot infer causality due to of the lack of temporal associations. Furthermore, caution should be used to extrapolate the findings from the ELANS data to rural populations or other countries of the region as this survey has only represented the dietary intake of the urban population of eight countries of LA. Although dietary data from the rural population were not investigated, one should notice that the majority of the LA population currently lives in an urban setting (64% to 92% of the population) [55].

## 5. Conclusions

Although the prevalence of breakfast consumption in LA was high, there were differences across the countries and specific groups. The main targets for encouraging breakfast intake are Brazil and Argentina, as well as adolescents, those who are underweight and those who are insufficiently physically active in LA. Overall, breakfast was an eating occasion rich in carbohydrate, added sugar, saturated fat and calcium relative to the entire day. The importance of regular breakfast consumption associated with balanced intake of nutrients should be part of the approach to improve compliance with nutritional recommendations and adherence to a healthy lifestyle. Findings from the present study will contribute to the development of data-driven nutrient recommendations for breakfast in LA.

## Figures and Tables

**Table 1:** Distribution of the ELANS (Latin American Study of Nutrition and Health/Estudio Latinoamericano de Nutrición y Salud) participants according to breakfast consumption habit, sex, age group, socioeconomic level, body weight status and physical activity and analyses of the factors associated with breakfast intake.

Category	Total (*n*)	Breakfast Classification						*p* *	Logistic Regression					
		Breakfast Skipper		Occasional Consumer		Regular Breakfast Consumer			Univariate Logistic Regression			Multiple Logistic Regression		
		*n*	%	*n*	%	*n*	%		OR	*p*	95% CI	OR	*p*	95% CI
**Sex**														
Male	4409	238	5.4	721	16.4	3450	78.3	0.534	reference			reference		
Female	4809	266	5.5	746	15.5	3797	79.0		0.97	0.089	[0.81–1.17]	0.96	0.914	[0.84–1.22]
**Age group**														
15–19 y	1223	83	6.8	235	19.2	905	74.0	***p* < 0.001**	reference			reference		
20–34 y	3479	189	5.4	618	17.8	2672	76.8		1.27	0.082	[0.97–1.66]	1.20	0.201	[0.91–1.60]
35–49 y	2627	143	5.4	385	14.7	2099	79.9		1.26	0.099	[0.96–1.67]	1.19	0.257	[0.88–1.62]
50–65 y	1889	89	4.7	229	12.1	1571	83.2		1.47	0.014	[1.08–2.00]	1.49	**0.021**	[1.06–2.10]
**Socioeconomic level**														
High	880	36	4.1	128	14.5	716	81.4	0.219	reference			reference		
Medium	3542	194	5.5	574	16.2	2774	78.3		0.74	0.099	[0.51–1.06]	0.75	0.129	[0.51–1.09]
Low	4796	274	5.7	765	16.0	3757	78.3		0.70	0.053	[0.49–1.00]	0.70	0.059	[0.49–1.01]
**Body weight status**														
Underweight	306	27	8.8	50	16.3	229	74.8	0.155	reference			reference		
Normal weight	3420	188	5.5	549	16.1	2683	78.5		0.60	0.018	[0.39--0.92]	0.63	**0.040**	[0.41–0.98]
Overweight	3167	165	5.2	483	15.3	2519	79.5		1.06	0.605	[0.85–1.31]	0.99	0.944	[0.79–1.25]
Obese	2315	124	5.4	384	16.6	1807	78.1		1.03	0.818	[0.81–1.30]	0.96	0.737	[0.75–1.23]
**Physical Activity**														
Insufficiently active	4196	256	6.1	667	15.9	3273	78.0	**0.024**	reference			reference		
Active	4636	223	4.8	728	15.7	3685	79.5		1.29	0.008	[1.07–1.55]	1.29	**0.007**	[1.07–1.55]

* Pearson chi-square test; OR, odds ratio; 95% CI, 95% confidence interval. Bold: significant difference accepted at *p* < 0.05. Multiple logistic regression models for breakfast consumption habit (skippers and regular/occasional consumers) were performed with three levels (age group, body weight status and physical activity), and adjusted for sex and socioeconomic level. Regular breakfast consumers: breakfast intake on both 24-HR; occasional breakfast consumers: breakfast intake in one 24-HR; and breakfast skippers: no breakfast intake on both 24-HR.

**Table 2:** Mean (SD) breakfast intake and contribution to daily intake (%) of energy, fiber and micronutrients of occasional (*n* = 1467) and regular (*n* = 7247) breakfast consumers, by country.

**Dietary Component**	**Country**														
	**Argentina**			**Brazil**			**Chile**			**Colombia**			**Costa Rica**		
	**Mean**	**SD**	**% DI**	**Mean**	**SD**	**% DI**	**Mean**	**SD**	**% DI**	**Mean**	**SD**	**% DI**	**Mean**	**SD**	**% DI**
Energy (kcal)	319	225	16	403	247	23	400	208	24	527	286	26	411	241	23
Carbohydrate (g)	60	37	22	57	34	26	59	31	26	71	41	25	61	35	23
Protein (g)	9	8	12	12	10	17	14	9	22	19	11	24	13	10	21
Fat (g)	8	9	11	14	13	24	12	9	23	20	15	28	14	11	25
Saturated fat (g)	4	5	13	5	5	27	6	4	27	8	6	31	5	4	27
Added sugar (g)	21	21	28	14	16	31	12	13	27	16	16	32	15	13	29
Dietary fiber (g)	2	2	16	3	2	22	3	2	21	4	3	24	5	4	27
Sodium (mg)	327	312	13	575	1089	21	553	357	23	530	411	29	616	457	23
Vitamin A (mcg)	67.8	167.9	13	133.7	171.2	35	123.9	154.4	26	171.8	266.7	32	186.1	223	33
Thiamin (B1) (mg)	0.4	0.3	22	0.4	0.2	27	0.4	0.3	27	0.5	0.3	31	0.5	0.3	27
Riboflavin (B2) (mg)	0.4	0.3	21	0.5	0.3	35	0.4	0.3	30	0.7	0.5	38	0.5	0.3	37
Niacin (B3) (mg)	2.8	2.7	14	3.4	2.6	19	4.2	3.4	22	5.4	3.8	23	4.1	2.8	22
Pyridoxine (B6) (mg)	0.2	0.2	12	0.2	0.2	13	0.2	0.4	16	0.4	0.3	18	0.3	0.3	17
Cobalamin (B12) (mcg)	0.4	0.7	12	0.6	0.7	21	0.7	1.1	22	1.4	2.4	31	0.7	0.9	20
Vitamin C (mg)	3.7	17.4	8	22.6	108.8	17	6.6	17.1	10	17.8	42.6	14	12.7	27.1	15
Vitamin D (mcg)	0.8	1.2	20	0.4	0.9	16	1.1	1.5	29	2.3	2	44	0.8	0.9	30
Calcium (mg)	129	151	18	168	163	36	170	170	31	323	230	43	129	129	28
Iron (mg)	2.4	2.1	17	2.5	1.8	26	3.3	2.7	26	3.9	3.1	27	3.6	2.5	26
Potassium (mg)	256	206	15	362	286	19	303	225	17	650	436	23	460	302	22
Magnesium (mg)	29.7	23.7	15	39.7	29.8	20	38.3	23.1	21	70.4	42.8	26	55.9	42.5	23
Zinc (mg)	4.8	5	22	1.5	1.9	16	1.7	1.7	20	2.5	1.8	23	1.7	1.4	20
**Dietary Component**	**Country**									**ELANS**					
	**Ecuador**			**Peru**			**Venezuela**								
	**Mean**	**SD**	**% DI**	**Mean**	**SD**	**% DI**	**Mean**	**SD**	**% DI**	**Mean**		**SD**		**% DI**	
Energy (kcal)	544	288	25	472	227	23	511	247	27	444		258		23	
Carbohydrate (g)	76	41	26	75	36	22	64	31	27	65		36		25	
Protein (g)	19	12	22	17	10	21	18	10	24	15		11		20	
Fat (g)	20	15	26	14	10	25	21	15	32	15		13		24	
Saturated fat (g)	7	6	30	4	4	28	7	5	33	6		5		27	
Added sugar (g)	16	10	36	18	13	30	17	14	30	16		15		30	
Dietary fiber (g)	4	3	23	4	3	22	4	3	29	3		3		23	
Sodium (mg)	968	733	20	395	264	39	879	437	31	589		656		25	
Vitamin A (mcg)	201.9	688.2	29	171.9	234.3	30	131.4	112.8	32	144.8		280.6		29	
Thiamin (B1) (mg)	0.5	0.3	27	0.4	0.3	24	0.7	0.3	35	0.5		0.3		27	
Riboflavin (B2) (mg)	0.5	0.4	35	0.4	0.3	31	0.6	0.3	36	0.5		0.3		33	
Niacin (B3) (mg)	5.4	3.8	21	4.7	3.0	19	6.1	3.2	26	4.4		3.3		20	
Pyridoxine (B6) (mg)	0.4	0.4	17	0.3	0.3	15	0.4	0.3	23	0.3		0.3		16	
Cobalamin (B12) (mcg)	1.4	5.0	31	0.7	1.3	21	1.1	1.4	30	0.8		2.0		23	
Vitamin C (mg)	24.2	43.3	16	21.0	45.3	18	30.6	49.6	21	18.0		60.2		15	
Vitamin D (mcg)	2.0	2.4	37	1.4	2.3	33	1.0	1.3	36	1.1		1.7		29	
Calcium (mg)	273	181	38	163	139	33	264	221	39	202		190		34	
Iron (mg)	3.6	2.5	24	3.6	2.1	27	4.0	2.5	30	3.3		2.5		25	
Potassium (mg)	648	499	23	474	335	21	448	305	23	441		357		20	
Magnesium (mg)	67.2	41.2	24	62.1	34.4	23	73.6	35.8	30	53.5		37.8		22	
Zinc (mg)	2.2	1.5	21	2.2	6.8	21	2.5	1.6	26	2.4		3.5		21	

SD, Standard Deviation. Regular breakfast consumers: breakfast intake on both 24-HR; occasional breakfast consumers: breakfast intake in one 24-HR.

## Supplementary Materials

The following are available online at https://www.mdpi.com/2072-6643/12/8/2397/s1, Figure S1: Flowchart of breakfast 24-HR selection.
