# Supplementary figures and images for: Breakfast Consumption Habit and Its Nutritional Contribution in Latin America: Results from the ELANS Study
Source: Nutrients. 2020 Aug 10;12(8):2397. doi: 10.3390/nu12082397 (PMC7468943; doi:10.3390/nu12082397)

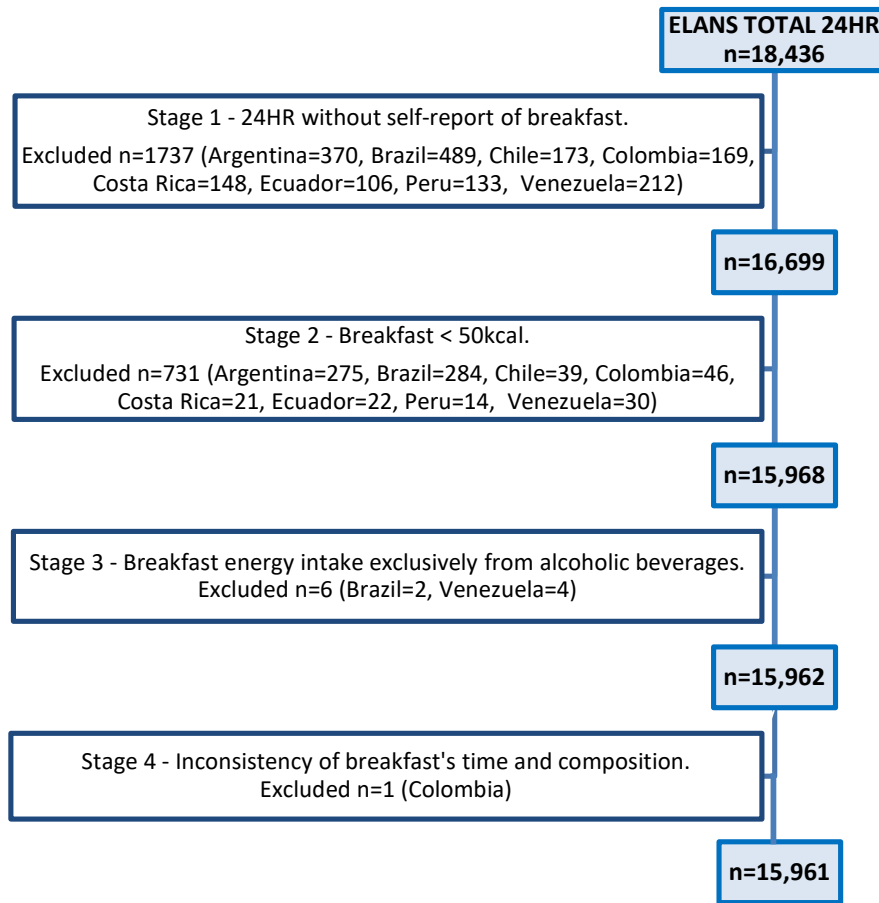

**Figure S1.** Flowchart of breakfast 24-HR selection. 24-HR reflects: 9218 individuals x 2 days of recall = 18,436 recalls.

Supplement: Supplementary file 1 [file nutrients-12-02397-s001.pdf]
